# Effects of Fatty Acid Oxidation and Its Regulation on Dendritic Cell-Mediated Immune Responses in Allergies: An Immunometabolism Perspective

**DOI:** 10.1155/2021/7483865

**Published:** 2021-08-11

**Authors:** Shanfeng Sun, Yanjun Gu, Junjuan Wang, Cheng Chen, Shiwen Han, Huilian Che

**Affiliations:** ^1^Key Laboratory of Precision Nutrition and Food Quality, Key Laboratory of Functional Dairy, Ministry of Education, Beijing 100083, China; ^2^College of Food Science and Nutritional Engineering, China Agricultural University, Beijing 100083, China

## Abstract

Type 1 allergies, involve a complex interaction between dendritic cells and other immune cells, are pathological type 2 inflammatory immune responses against harmless allergens. Activated dendritic cells undergo extensive phenotypic and functional changes to exert their functions. The activation, differentiation, proliferation, migration, and mounting of effector reactions require metabolic reprogramming. Dendritic cells are important upstream mediators of allergic responses and are therefore an important effector of allergies. Hence, a better understanding of the underlying metabolic mechanisms of functional changes that promote allergic responses of dendritic cells could improve the prevention and treatment of allergies. Metabolic changes related to dendritic cell activation have been extensively studied. This review briefly outlines the basis of fatty acid oxidation and its association with dendritic cell immune responses. The relationship between immune metabolism and effector function of dendritic cells related to allergic diseases can better explain the induction and maintenance of allergic responses. Further investigations are warranted to improve our understanding of disease pathology and enable new treatment strategies.

## 1. Introduction

Allergic diseases are an increasing health challenge worldwide. In developed countries, IgE-mediated type 1 hypersensitivity disorders have been reported to affect more than 25% of the population [1]. Immunologically, the occurrence and maintenance of type 1 allergies are caused by exaggerated type 2-mediated immune responses against harmless antigens. The hallmark characteristics of allergic inflammation are strong bronchoconstriction/persistent bronchospasm, immune cell recruitment, airway inflammation, hyperresponsiveness, and tissue remodeling. The main effector cells involved in the establishment and initiation of allergic responses are antigen-presenting cells (dendritic cells (DCs) and macrophages), T helper 2 (Th2) cells, plasma cells producing IgE, eosinophils, basophils, mast cells, and innate lymphocyte type 2 cells (ILC2s).

DCs, key mediators of the immune response [2], are the most powerful professional antigen-presenting cells in the body and can efficiently ingest, process, and present antigens. They are the only antigen-presenting cells that can activate nonsensitized naive T cells and are central to the initiation, regulation, and maintenance of the immune response. DCs are known to regulate upstream processes in allergic responses and act as a bridge between the antigen and T cells. Therefore, it is of great significance to understand the role of immune metabolism changes in DCs in these reactions to better understand the molecular mechanisms underlying allergic responses in order to develop novel strategies for the treatment and prevention of these conditions.

Activated immune cells undergo extensive phenotypic and functional changes to exert their function. The activation, differentiation, proliferation, migration, and mounting of effector reactions require metabolic reprogramming [1]. Current research has shown that DCs undergo distinct metabolic changes upon activation [3]. “Immune metabolism”, a new research field, can explain the potential mechanism of metabolic changes and their functional consequences [4].

Fatty acid oxidation (FAO) is central to various physiological and pathological processes, including allergies [5], obesity [6], cancer [7], and nonalcoholic fatty liver disease (NAFLD) [8]. FAO reprogramming plays an important role in maintaining and establishing the phenotype and function of immune cells, which include DCs [9], macrophages [10], CD4^+^ T [11], and ILC2s [12]. This review briefly introduces the fundamental principle of cellular FAO and its relationship with DC function and discusses the metabolic adaptation associated with the activation of DCs in the context of allergies.

## 2. FAO and Main Enzyme Markers

**Figure 1:**
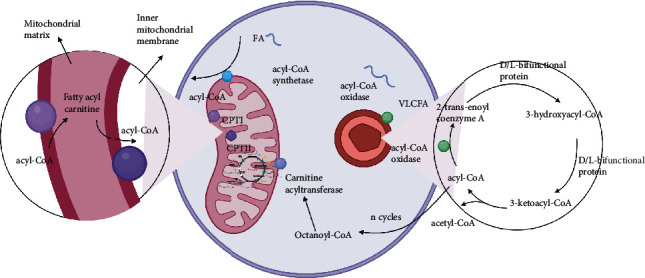
Oxidation of fatty acids in the mitochondria and peroxisomes. VLCFA: very long-chain fatty acid (22 or more carbons); FA: fatty acid (less than 20 carbons); CPT I: carnitine palmitoyltransferase I; CPT II: carnitine palmitoyltransferase II.

The mitochondria are the primary sites of FAO and use this process to create acetyl-CoA. After entering cells, fatty acids are activated by the enzyme acyl-CoA synthetase located in the outer membrane of the mitochondria to produce acyl-CoA, which is then transported into the mitochondria to facilitate further oxidative metabolism (Figure 1). The mitochondria primarily use ≤20 carbon atoms for this process, and this oxidation pathway usually includes the following three steps: (1) cells ingest fatty acids, which undergo activation to produce acyl-CoA; (2) acyl-CoA is transported into the mitochondria; (3) undergoes *β*-oxidation to produce acetyl-CoA. Short- or medium-length acyl-CoA molecules (<10 carbon atoms) can easily penetrate the inner mitochondrial membrane, but longer-chain acyl-CoA molecules must be transported into the mitochondria via the enzyme carnitine acyltransferase system. This system predominantly relies on the activity of three enzymes, namely, carnitine palmitoyltransferase I (CPT I), carnitine/acylcarnitine transferase, and carnitine palmitoyltransferase II (CPT II). Long-chain fatty acids are combined with carnitine molecules under the catalysis of CPT I to generate fatty acyl carnitine, which is transported into the inner mitochondrial membrane by the enzyme carnitine acyl carnitine translocase. After entering the mitochondrial matrix, free carnitine molecules are released by CPT II, and acyl-CoA is resynthesized allowing further oxidative metabolism.

The peroxisome is a subcellular organelle found in most organs and acts as a secondary site for FAO [13]. These oxidative reactions commonly follow four reaction processes: (1) oxidation: dehydrogenation of acyl-CoA by the enzyme acyl-CoA oxidase produces 2-trans-enoyl coenzyme A; (2) a hydration reaction, where 2-trans-enoyl-CoA is converted into 3-hydroxyacyl-CoA by *D*-bifunctional protein or *L*-bifunctional protein following the addition of water; (3) dehydrogenation reaction, where 3-hydroxyacyl-CoA is dehydrogenated into 3-ketoacyl-CoA using the same bifunctional protein as in step 2; (4) thiolysis reaction, where 3-ketoacyl-CoA thiolase converts 3-ketoacyl-CoA into 1 molecule of acetyl-CoA (or propionyl-CoA) and acyl-CoA with a shortened carbon chain, which can then enter the next cycle (Figure 1) [14]. There are several similarities between peroxisomes and mitochondrial oxidation but also several important differences such as (1) during the initial step of oxidation in peroxisomes, acetyl-CoA oxidase generates hydrogen peroxide instead of NAD; (2) the *β*-oxidation process in peroxisomes consumes less energy and releases more heat than that in the mitochondria; (3) peroxisomes do not include the proteins of the respiratory chain and thus do not generate ATP, meaning that the *β*-oxidation of fatty acids in peroxisomes is not limited by cellular energy requirements [15]. Based on this, the *β*-oxidative metabolism of peroxisomes is more suited for the partial oxidation of fatty acids and catalysis of some exogenous complexes that cannot be oxidized by mitochondrial enzymes. Notably, after long-chain fatty acids undergo one or more limited *β*-oxidation cycles in peroxisomes, their carbon chains gradually shorten, thereby creating medium- and short-chain fatty acyl-CoA, suggesting that this oxidation process is incomplete. These products are eventually transported into the mitochondria and used to synthesize acetyl-CoA, which then enters the tricarboxylic acid cycle (TCA) and oxidative phosphorylation (OXPHOS) pathways, ultimately producing carbon dioxide, water, and a large amount of ATP. Furthermore, FAO products may also act as signaling molecules that can directly regulate cellular homoeostasis by modulating the surrounding microenvironment to create conditions conducive to pathological progression [16].

## 3. Signal Pathways of Regulating FAO in DCs

FAO regulation is complex since it involves the activity of several different enzymes depending on the substrate involved. Among them, CPT I and ACOX are the key enzymes for FAO in the mitochondria and peroxisomes, respectively. These enzymes are regulated by various signaling pathways, making these pathways indirect regulators of FAO. These pathways include the adenosine 5′-monophosphate-activated protein kinase (AMPK) pathway, the peroxisome proliferator-activated receptor (PPAR) pathway, the signal transducer and activator of transcription 3 (STAT3) pathway, and the peroxisome proliferator-activated receptor gamma coactivator 1 (PGC-1) pathway.

### 3.1. Effect of the AMPK Signaling Pathway on FAO in DCs

AMPK is a highly conserved serine/threonine protein kinase commonly found in eukaryotic cells, as a heterotrimer (*αβγ*), and immune cells, such as DCs, macrophages, lymphocytes, and neutrophils, and is an important regulator of inflammatory responses through the regulation of complex signaling networks in part by inhibiting downstream cascade pathways, such as NF-*κ*B, which is a key regulator of innate immunity and inflammation, and acts as a negative regulator of Toll-like receptors (TLRs) [17]. AMPK regulates ATP production and consumption in eukaryotic cells, making it a key regulator of bioenergy metabolism. Several upstream kinases, including transient receptor potential vanilloid-1 (TRPV1), liver kinase B1 (LKB1), calcium/calmodulin kinase *β* (CaMK *β*), and TGF-*β*-activated kinase 1 (TAK-1), activate AMPK by phosphorylating threonine residues at its *α*-catalytic subunit. When cell energy metabolism is impaired, the intracellular AMP/ATP ratio increases, thereby activating AMPK, which in turn inhibits various energy-consuming biosynthesis pathways, including the protein, fatty acids, and glycogen synthesis pathways, and activates several catabolic pathways, including FAO (activated AMPK inhibit CPT I by inhibiting the enzyme ACC1, ACC2, or malonyl-CoA decarboxylase, ultimately increasing FAO), to produce ATP [18]. Immunologically, the AMPK-mediated FAO affects DC differentiation. Kratchmarov et al. reported that phosphorylation of AMPK itself as well as the AMPK targets ACC increased the development of cDC2 cells at the expense of cDC1 cells [19]. In contrast, activated AMPK can inhibit the maturation of DCs and induce immune tolerance. Yao et al. reported that oleoylethanolamide (OEA), an endogenous lipid mediator, downregulates TLR4/NF-*κ*B, the classical pathway leading to DC maturation induced by lipopolysaccharide (LPS), through the activation of TRPV1 and AMPK. OEA treatment decreases the expression of cell surface markers, reduces cell migration, diminishes the proliferation of cocultured T cells, and regulates cytokine production in bone marrow-derived DCs (BMDCs) induced by LPS, which illustrate the inhibitory effect of OEA on DC maturation [20].

### 3.2. Effect of the PPAR Signaling Pathway on FAO in DCs

PPARs are ligand-activated transcription factors that regulate genes important for cell differentiation and various metabolic processes, especially lipid and glucose homeostasis [21]. PPARs have three subtypes, namely, *α*, *β*/*δ*, and *γ*, which show different expression patterns in different vertebrates and cells. In humans, the following genes encode three types of PPARs: PPARA-*α*, PPARD-*β*/*δ*, and PPARG-*γ*. The expression profiles of PPARs in peripheral blood DCs are listed in Table 1, which shows that PPAR subunits are all expressed in peripheral blood DCs and may activate FAO and induce immune dysfunctional DCs [22]. PPAR-*α* plays a role in scavenging circulating and intracellular lipids by regulating the expression of FAO-related genes. PPAR-*β*/*δ* is involved in lipid oxidation and cellular proliferation, while PPAR-*γ* promotes adipocyte differentiation, indirectly increasing glucose uptake [21]. All PPARs form heterodimers with retinol-like X receptor (RXR) and bind to specific regions of the target gene DNA, known as peroxisome proliferator hormone response elements (PPREs) [23]. PPAR-*α* is a key regulator of FAO and is a target of fibrates in the treatment of human lipid diseases [24, 25]. The functions of PPAR-*δ* and PPAR-*α* overlap, yet PPAR-*δ* is more widely expressed [26]. PPAR-*γ* is a target of glitazone drugs for the treatment of human diabetes, and its expression is enriched in adipose tissues where it is essential for adipogenesis [27, 28]. Huang et al. showed that PARP1 is a transcriptional repressor of the PPAR-*α* gene, and its activation inhibits the transactivation of PPAR-*α* and target gene expression induced by its ligands (fenofibrate). PPAR-*α* is also a substrate of PARP1-mediated poly-ADP ribosyl. This poly-ADP ribosylation of PPAR-*α* inhibits its recruitment to target gene promoters and its interaction with SIRT1 (a key regulator of PPAR-*α* signal transduction), thus inhibiting fatty acid-induced upregulation of FAO [8]. Increased tolerance in local DCs in the tumor microenvironment may promote immune escape; melanoma is known to induce FAO in DCs via the Wnt5a-*β*-catenin-PPAR-*γ* signaling pathway and upregulate CPT1A protein expression. Elevated FAO levels increase the protoporphyrin IX prosthetic group of indoleamine 2,3-dioxygenase-1 (IDO) expression and downregulate IL-6 and IL-12 cytokine expression, thereby enhancing IDO activity and increasing the production of regulatory T cells [29]. Moreover, PPAR-*γ*-deficient DCs showed enhanced immunogenicity and could induce tolerant T cell responses [11], which further supports the relationship between FAO and the immune-suppressive phenotype.

### 3.3. Effect of the STAT3 Signaling Pathway on FAO in DCs

STAT3 is a transcription factor encoded by the human STAT3 gene [30], and is an active member of the STAT protein family. STAT3 is phosphorylated by receptor-related Janus kinase (JAK), following the expression of specific cytokines and growth factors, allowing for the production of various homo- or heterodimers, which are transferred to the nucleus where they act as transcriptional activators. Interferon, epidermal growth factor, interleukin (IL-5 and IL-6), and several other ligands act on the receptor, resulting in the phosphorylation of tyrosine 705 in the STAT3 protein and its activation. In addition, STAT3 may be activated following the phosphorylation of serine 727 by mitogen-activated protein kinase [31] and c-Src nonreceptor tyrosine kinase [32, 33]. STAT3 mediates the expression of various genes in response to various stimuli and plays a key role in many cellular processes, including growth and apoptosis [34]. Activation of the second messenger JNK can phosphorylate STAT3, and phosphorylated STAT3 then acts on CPT I to increase FAO [7].

### 3.4. Effect of the PGC-1 Signaling Pathway on FAO in DCs

The PGC-1 coactivator family comprises three different members, namely, PGC-1*α*, PGC-1*β*, and PGC-1 related coactivator (PRC). PGC-1*α* regulates thermogenesis by interacting with PPAR-*γ* in brown adipose tissue [35], while the remaining two are described using sequence homology against PGC-1*α* [36, 37]. Current studies have found that PGC-1*α* and PGC-1*β* are related to FAO [38, 39]. Kleiner and colleagues found that PGC-1*α*, but not PGC-1*β*, is essential for the full activation of key lipid metabolism genes (such as CPT-1B) via PPAR-*δ* agonism. Furthermore, the induction of FAO by PPAR-*δ* agonism was completely abolished during the absence of both PGC-1*α* and PGC-1*β*. Conversely, PGC-1*α*-driven FAO is independent of PPAR-*δ*. These results demonstrate that the pharmacological activation of PPAR-*δ* induces FAO via PGC-1*α*. However, PGC-1*α*-induced FAO appears to be independent of PPAR-*δ* [38]. In another study, knockdown of PGC-1*β* resulted in the inhibition of FAO and anti-inflammatory functions [39]. In contrast to PGC-1*α* and PGC-1*β*, more studies are warranted to determine whether PRC plays a role in FAO.

## 4. Effect of FAO on Immune Metabolism of DCs

### 4.1. FAO Affects OXPHOS Metabolism of DCs

**Figure 2:**
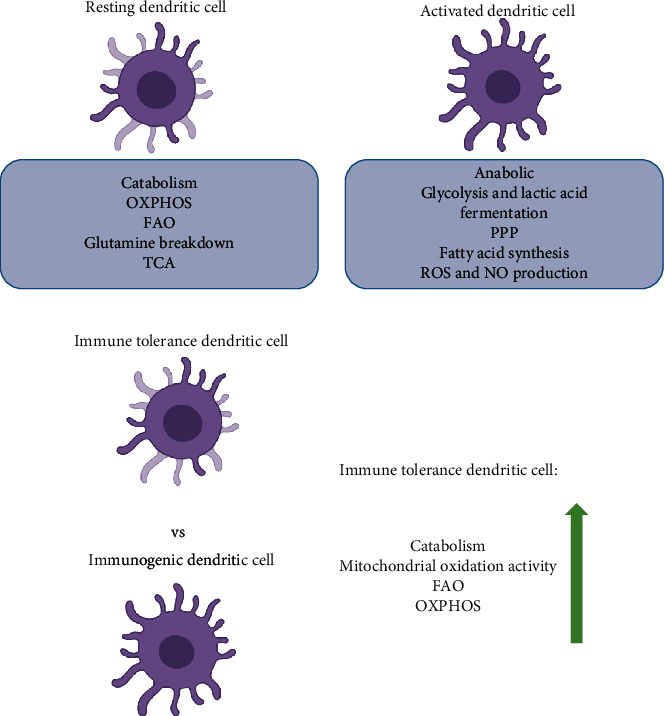
Metabolism of different types of DCs during different metabolic states. (a) Resting and activated. (b) Immune tolerance vs. immunogenic. OXPHOS: oxidative phosphorylation; FAO: fatty acid oxidation; TCA: tricarboxylic acid cycle; PPP: pentose phosphate pathway.

The OXPHOS pathway plays an important role in FAO metabolism. OXPHOS accepts electrons from acetyl CoA dehydrogenase and is known to work in concert with FAO during various physiological and pathological processes. Lin and colleagues found that FAO promotes cellular reprogramming by enhancing OXPHOS and inhibiting protein kinase C activity during the early stages of reprogramming [40]. The mitochondria are the main energy source in eukaryotic cells that oxidize both fats and carbohydrates to produce ATP. Mitochondrial FAO and OXPHOS are the two key metabolic pathways involved in this process. There are several physical interactions between FAO and OXPHOS proteins; all of which are crucial for the function of both FAO and OXPHOS [41]. OXPHOS acts downstream of FAO, and these two pathways work together to inhibit or promote the initiation and progression of different immune responses in DCs. For example, resting DCs generate energy through OXPHOS and FAO, whereas activated DCs rapidly shift to the glycolytic pathway. Resting DCs exhibited high rates of catabolism and constantly decomposed nutrients to generate energy to maintain cell survival (Figure 2(a)). This metabolic state demonstrates active OXPHOS, which is driven by the TCA cycle, fueled by FAO and glutamine decomposition products, and regulated by AMPK/PGC-1*α* [3, 42–46].

**Figure 3:**
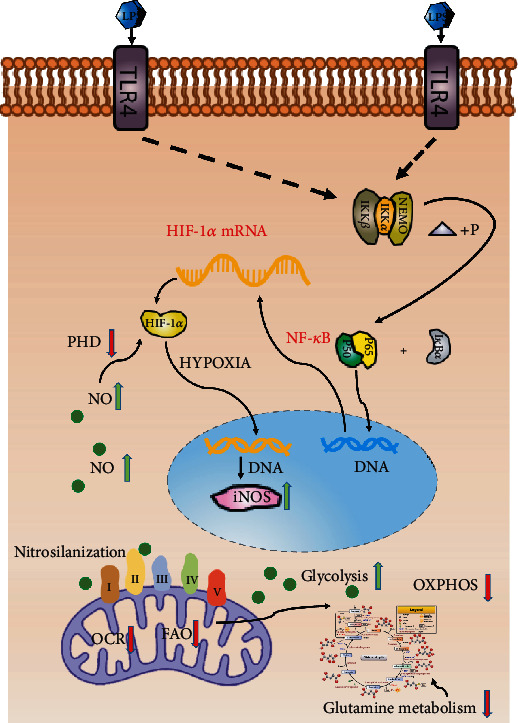
NO was induced in DCs following LPS stimulation. When OXPHOS decreased, this enhanced glycolysis rate became NO dependent. Stable HIF-1*α* enhances NO production by increasing the expression of iNOS, which leads to the inhibition of prolyl hydrolase, a marker for HIF-1*α* degradation. This positive feedback loop results in the accumulation of NO, which leads to the nitrosilanization of some ETC complexes and the inhibition of their function. NF-*κ*B: nuclear factor kappa-light-chain-enhancer of activated B cells; IKK*β*: inhibitory *κ*B kinase *β*; IKK*α*: inhibitory *κ*B kinase *α*; NEMO: NF-*κ*B essential modifier; PHD: prolyl hydrolase; I*κ*B*α*: inhibitors of NF-*κ*B *α*.

After DC activation, anabolism is often used to produce substrates for biosynthesis and cell growth. These activated DCs use glycolysis and lactic acid fermentation instead of FAO and OXPHOS to meet the energy demands of the cell, with the glycolysis intermediates being reintroduced into the pentose phosphate pathway. Meanwhile, DCs were transformed from immune tolerance to immunogenicity (Figure 2(b)). TCA is redistributed, resulting in the accumulation of TCA intermediates, which can be used as immunomodulatory signals and support fatty acid synthesis and production of ROS and nitric oxide (NO) during DC activation [3, 43, 45] (Figure 2(a)). NO production mediated by inducible nitric oxide synthase (iNOS) is the key to the effector functions of activated granulocyte-macrophage colony-stimulating factor-induced DCs (GM-DCs) and the metabolic collapse of OXPHOS [47]. Within 6 h of LPS stimulation, GM-DCs induced NO production, decreased OXPHOS, and increased glycolysis. Approximately 9 h poststimulation, the enhanced glycolysis becomes NO-dependent [48] and HIF-1*α* enhances NO production by upregulating iNOS, which in turn leads to the inhibition of prolyl hydrolase, which degrades HIF-1*α*. This positive feedback loop results in the accumulation of NO, leading to nitrosilanization of some of the ETC complexes, resulting in their loss of function, thereby reducing FAO and OXPHOS [47, 49, 50] (Figure 3). In DCs, HIF-1*α* deficiency decreased the expression of MHC-II, CD80, and CD86, resulting in impaired T cell stimulatory capacity [51, 52]. Mouse moDCs induced by *Listeria monocytogenes* also demonstrated similar NO-mediated oxygen consumption rate (OCR) inhibition in the late stages of stimulation, which could be compensated by enhanced glycolysis [47]. LPS can also rapidly induce glycolysis in DCs by activating TANK-binding kinase 1 and/or NF-*κ*B kinase *ε* and inhibitors of hexokinase 2 in an HIF-1*α*-independent manner [53].

### 4.2. FAO Affects Immune Responses of DCs

Contrary to the proinflammatory characteristics of immunogenic DCs, immunotolerant DCs show immune nonresponse. Compared with immunogenic DCs, immunotolerant DCs showed significantly increased catabolic activity related to FAO, OXPHOS, and mitochondrial oxidation activity (Figure 2(b)). In addition, ETC complexes were more abundant in immunotolerant DCs. The inhibition of FAO inhibited immunotolerant DC function and partially restored their ability to stimulate T cell activation, suggesting that immunotolerant DCs are dependent on the FAO pathway for their phenotype [54].

However, the importance and function of FAO, mitochondrial energy metabolism, OCR, and OXPHOS in immunogenic DCs remain nebulous, and these outcomes appear to be tightly linked to the environment and DC subset. In some cases, the production of mitochondrial energy contributes to the activation of DCs, whereas in others, it does not. For example, Lawless et al. showed a contrasting function for glucose in DCs, since glucose represses the proinflammatory output of LPS-stimulated DCs and inhibits DC-induced T cell responses. A glucose-sensitive signal transduction circuit involving mTOR complex 1, HIF1*α*, and iNOS coordinates DC metabolism and functions to limit DC-stimulated T cell responses. However, when multiple T cells interact with DCs, they compete for nutrients, which can limit the glucose availability to the DCs. In such DCs, glucose-dependent signaling is inhibited, altering DC outputs and enhancing T cell responses [49].

**Figure 4:**
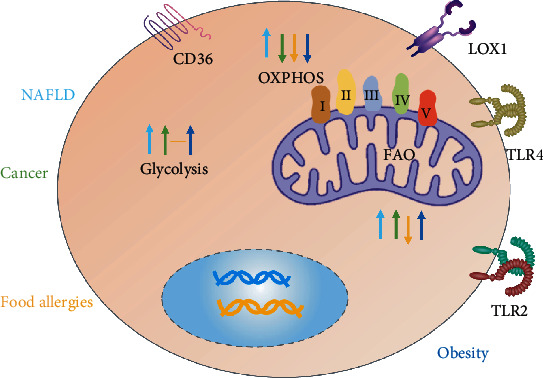
DCs in different pathological microenvironments have different FAO and metabolic status [6, 8, 59, 60, 62, 63].

Therefore, future studies should determine the impact of the microenvironment on DC activation and phenotype. This should not be limited to nutrients or oxygen supply, but should include other environmental factors that may strongly affect DC metabolism, such as extracellular levels of lactic acid, fatty acids [3], TCA intermediates (such as succinic acid and fumaric acid) [34, 45], and IL-10 [42, 55] or IFN-1 [56–58]. In addition, different pathological microenvironments (allergies, tumors, NAFLD, obesity, and glucose intolerance) where immune responses are initiated and maintained can change the metabolism; therefore, the functional properties of DCs should also be considered. The current study suggests that FAO is decreased during allergies [59, 60]. Emerging evidence indicates that the tolerization of local DCs that promote immune evasion within the tumor microenvironment is associated with the upregulation of FAO [29, 61]. Fatty acids can upregulate FAO in NAFLD [8]. In chronic high-fat diet mice, FAO inhibition induces a systemic hormetic response that protects mice from HFD-induced obesity and glucose intolerance [6]. The microenvironment influence on FAO in DCs should also be considered (Figure 4).

## 5. The Regulation of FAO on DC-Mediated Immune Responses in Allergies

An increasing number of studies have focused on the influence of FAO on the immune responses of DCs during allergies [20, 64–68]. The role of PPAR-*γ* in allergies has been studied extensively. FAO plays a role in DC-driven Th2 polarization, as shown by previous studies in which PPAR-*γ* was targeted in DCs [64, 65, 69]. It has been demonstrated that sirtuin 1 represses the activity of the nuclear receptor PPAR-*γ* in DCs, thereby favoring their maturation toward a pro-Th2 phenotype [64]. Hammad et al. reported that the activation of PPAR-*γ* alters the maturation process of DCs. The authors investigated the possibility that PPAR-*γ* activation, by targeting DCs, may be involved in the regulation of the pulmonary immune response to allergens. The activation of PPAR-*γ* prevents the induction of Th2-dependent eosinophilic airway inflammation and may contribute to immune homeostasis in the lungs [65]. Moreover, L. gasseri prevents mite allergen (Der p)-induced airway inflammation via activation of PPAR-*γ* in DCs. L. gasseri-induced PPAR-*γ* activation inhibits the development of airway inflammation in WT and PPAR-*γ*(+/-) mice [66]. In addition, Gilles et al. reported that pollen-derived E1-phytoprostanes modulate DC function via PPAR-*γ*-dependent pathways which inhibits NF-*κ*B activation thereby reducing IL-12 production by DCs and consecutive Th2 polarization [70]. Consistent with these findings, Hammad et al. showed that the activation of PPAR-*γ* in DCs inhibits the development of eosinophilic airway inflammation in a mouse model of asthma. However, this effect seems to be secondary to the induction of regulatory T cells, instead of the inability to induce Th2 responses when PPAR-*γ* is activated [65, 69]. These findings suggest that the activation of PPAR-*γ* increases FAO, which makes DCs present a tolerance phenotype. Taken together, these findings suggest that PPARs integrate FAO into the immune response during allergies.

Besides PPAR-*γ*, the STAT3-mediated FAO targeted in DCs also plays an important role in allergies. Xue et al. found that extracts of Bifidobacterium infantis (EBI) induced IL-10-producing DCs by increasing IL-35 and STAT3 phosphorylation. IL-10-expressing DCs induced IL-10-producing B cells, with the latter showing immunosuppressive functions. EBI significantly suppressed the levels of Th2 cytokines (IL-4, IL-5, and IL-13), which suggests that EBI has translational potential for use in the treatment of allergic diseases [67]. Consistent with this, Kitamura et al. showed that IL-6-STAT3-mediated increase in cathepsin S activity reduces the MHCII *αβ* dimer, Ii, and H2-DM levels in DCs and suppressed CD4^+^ T cell-mediated immune responses [68].

AMPK signal is associated with DC induction of Th2 cell differentiation [71]. AMPK promotes FAO, mitochondrial OXPHOS, and other forms of catabolic metabolism to generate more ATP during conditions of decreased ATP availability [72]. Upon LPS stimulation, mouse BMDCs show a rapid dephosphorylation of AMPK, followed by a decrease in FAO, which increased costimulatory molecule expression, while AMPK agonists decreased DC maturation [42]. Similarly, increased Ca^2+^ activates AMPK, which suppresses the proinflammatory responses of DCs [73]. Furthermore, Nieves and colleagues reported that defective AMPK activity in myeloid DCs negatively impacts type 2 responses through increased IL-12/23p40 production [71]. Elesela et al. found that sirtuin 1 activated AMPK, resulting in ACC-mediated stimulation of FAO *in vitro*. This process altered the T cell differentiation induced by the respective DCs from Th2/Th17 towards Th1 responses both *in vitro* and *in vivo*, suggesting that AMPK-regulated FAO metabolism plays an important role in DC function [74]. Above all, AMPK-mediated FAO can help to regulate the immune response of DCs in allergic diseases.

For PGC-1, although the regulation of PGC-1 on FAO has been reported, studies on the contribution of PGC-1-mediated FAO to DC immunometabolism in allergic diseases remain limited, and hence, further investigations are warranted.

Taken together, FAO impacts DC function and fate, which also influences the induction and maintenance of allergic reactions.

## 6. Conclusion

Type 1 allergies result from complex interactions between DCs and other immune cells. In order to exert their function, DCs change their metabolic phenotype during activation, from high rates of catabolism (OXPHOS, FAO, glutamine breakdown, and TCA) during the resting state to high rates of anabolism (glycolysis and lactic acid, fermentation, PPP, fatty acid synthesis, ROS, and NO production) during the activated state. In addition to the inherent changes in the metabolic phenotype of DCs, the local microenvironment of initiating and maintaining immune responses can also change the metabolism of DCs, thus changing their functional characteristics. Therefore, research on the immunometabolism of DCs in allergic diseases is important. Understanding the FAO metabolic changes related to the activation of DCs involved in the induction and maintenance of allergic reactions may lead to a more comprehensive understanding of the disease pathology and identification of novel target molecules and strategies for treating allergic diseases. AMPK, PPAR-*γ*, STAT3, and PGC-1 in DCs have been identified as potential targets for regulating allergic responses using immunometabolic analysis of FAO. Further studies can improve our understanding of the complex connections between FAO and immune responses of DCs in allergies.

## Figures and Tables

**Table 1:** The expression profiles of PPARs in peripheral blood DCs.

PPAR subunits	Gene names	RNA expression in plasmacytoid DC (NX)	RNA expression in myeloid DC (NX)
*α*	PPARA	3.8	0.7
*β*/*δ*	PPARD	1.8	2.9
*γ*	PPARG	0.0	0.4

NX: normalized expression. Data from the Human Protein Atlas (https://www.proteinatlas.org/).

## Supplementary Materials

Supplementary Materials Graphical abstract. The increase of fatty acid oxidation signal pathways (AMPK, PPAR-*γ*, PGC-1, and STAT3) in dendritic cells decreased the type 2 inflammatory immune responses in allergies.
