# Supplementary figures and images for: Effects of Fatty Acid Oxidation and Its Regulation on Dendritic Cell-Mediated Immune Responses in Allergies: An Immunometabolism Perspective
Source: J Immunol Res. 2021 Aug 11;2021:7483865. doi: 10.1155/2021/7483865 (PMC8376428; doi:10.1155/2021/7483865)

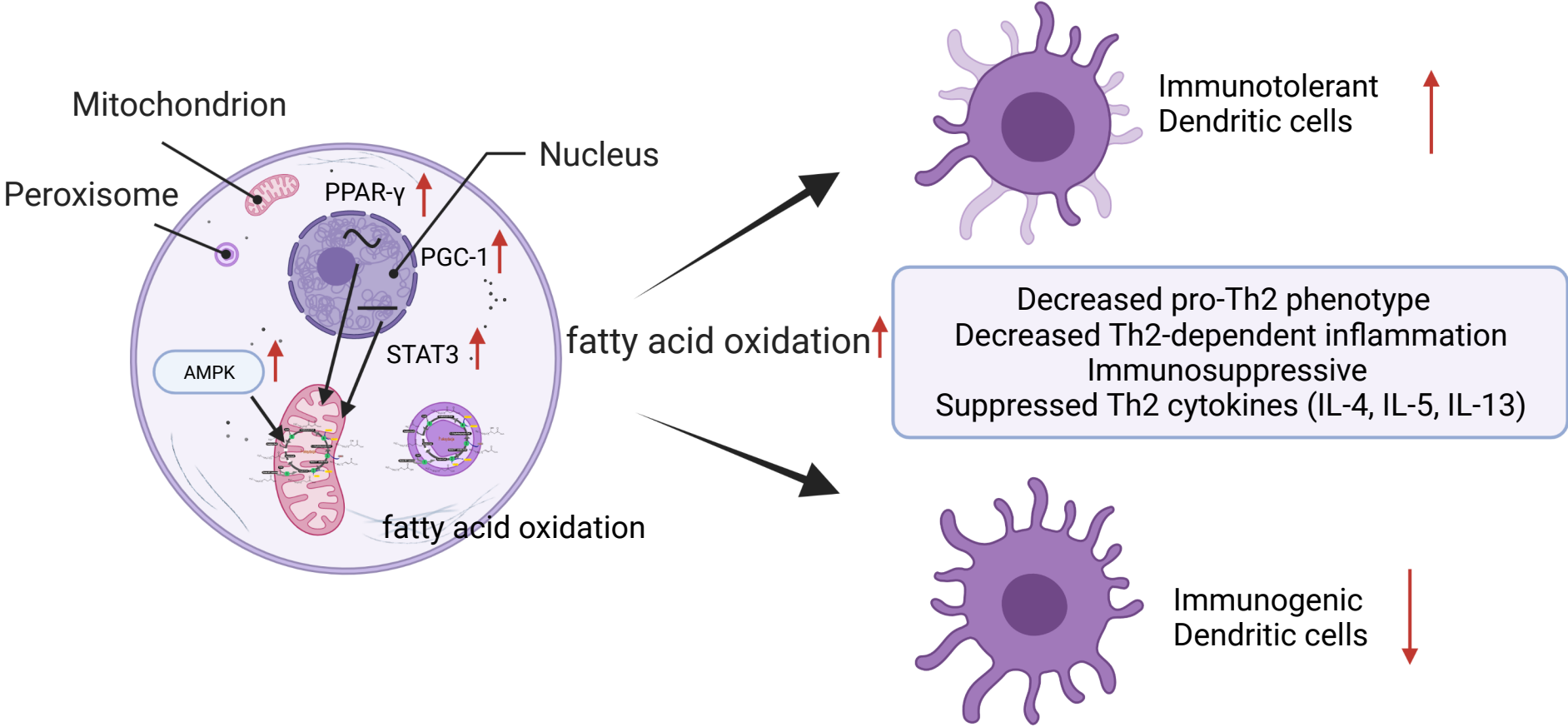

Supplement: Supplementary Materials — Graphical abstract. The increase of fatty acid oxidation signal pathways (AMPK, PPAR-γ, PGC-1, and STAT3) in dendritic cells decreased the type 2 inflammatory immune responses in allergies. [file 7483865.f1.pdf]
